# Supplementary material for: A domesticated prophage enzyme links bacterial metabolism, episymbiosis and phage fitness
Source: bioRxiv. 2026 Oct 9:2026.10.08.757619. Preprint. [Version 1] doi: 10.64898/2026.10.08.757619 (PMC13692244; doi:10.64898/2026.10.08.757619)
Supplement: Supplementary file 1 [file NIHPP2026.10.08.757619v1-supplement-1.pdf]

bioRxiv preprint doi: https://doi.org/10.64898/2026.10.08.757619; this version posted October 10, 2026. The copyright holder for this preprint (which was not certified by peer review) is the author/funder, who has granted bioRxiv a license to display the preprint in perpetuity. It is made available under a CC-BY 4.0 International license.

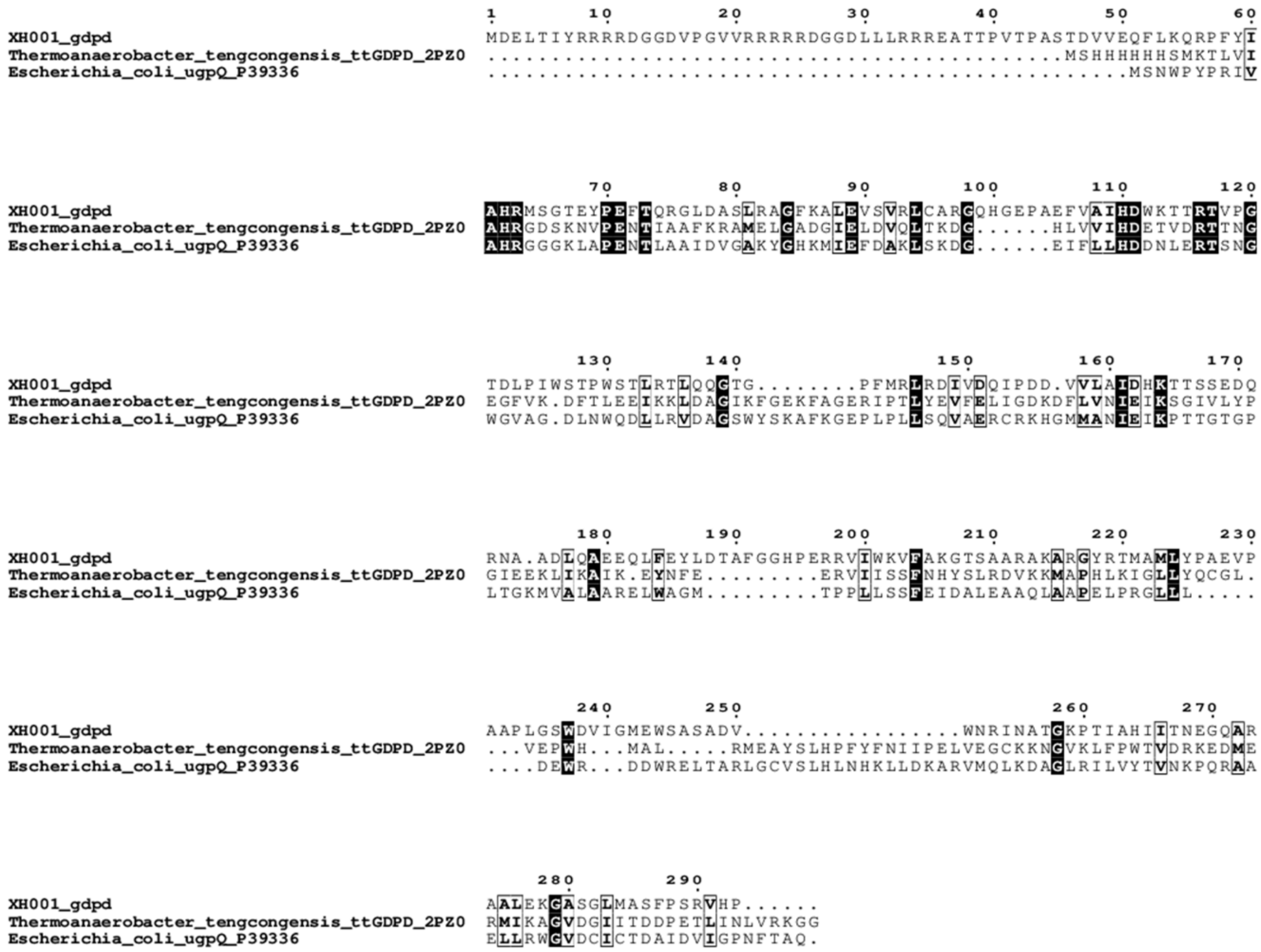

Supplementary Figure 1: Multiple sequence alignment of GDPD$_{Xhp1}$ enzymes. Multiple sequence alignment (MSA) of the prophage-encoded glycerophosphodiesterase GDPD$_{Xhp1}$ with experimentally characterized homologues from *T. tengcongensis* (ttGDPD, PDB ID: 2PZ0) and *E. coli* (ugpQ, UniProt ID: P39336). The alignment was generated using Clustal Omega and formatted using ESPript 3.0. Identical residues across all three sequences are highlighted in black boxes with white text. Conserved residues with similar physicochemical properties are enclosed in thin black outlines. Dots (.) represent alignment gaps introduced to maximize sequence homology. Numbering at the top corresponds to the total alignment position.

bioRxiv preprint doi: https://doi.org/10.64898/2026.10.08.757619; this version posted October 10, 2026. The copyright holder for this preprint (which was not certified by peer review) is the author/funder, who has granted bioRxiv a license to display the preprint in perpetuity. It is made available under a CC-BY 4.0 International license.

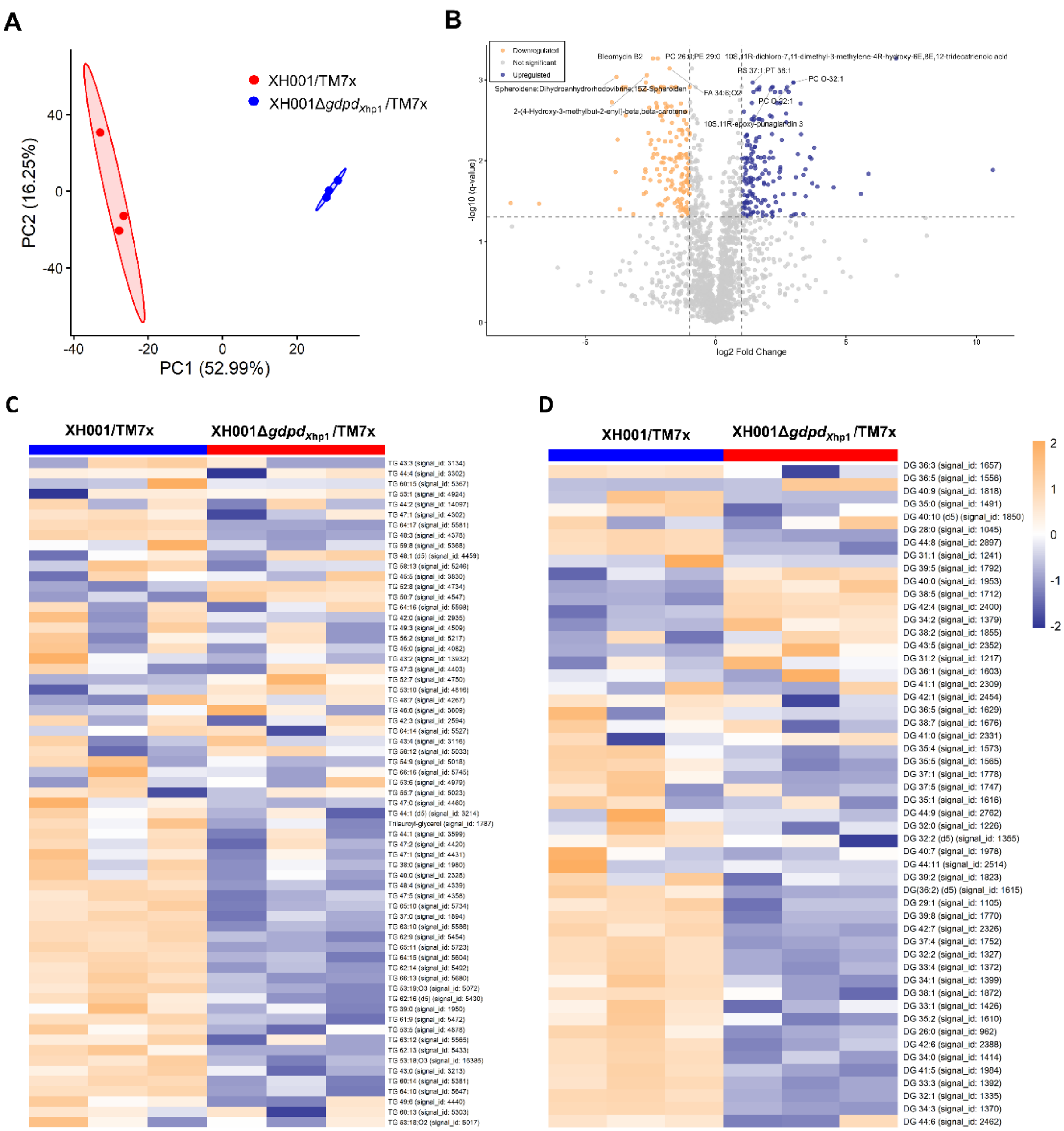

Supplementary Figure 2: Global lipidomic profiling reveals extensive remodeling of neutral lipid metabolism in XH001/TM7x coculture. (A) PCA of lipidomic profiles demonstrating clear separation between XH001/TM7x and XH001Δ*gdpd*$_{Xhp1}$/TM7x cocultures, indicating distinct lipid compositions. Ellipses represent the 95% confidence regions for each group. (B) Volcano plot showing differential lipid metabolite abundance in XH001Δ *gdpd*$_{Xhp1}$/TM7x relative to XH001/TM7x. Log_2 fold changes are plotted against adjusted *P* values (q values). Significantly decreased and increased metabolites are highlighted in orange and blue, respectively. (C-D) Heatmaps of triacylglycerols (GL0301) (C) and diacylglycerols (GL0201) (D). Each row represents

bioRxiv preprint doi: https://doi.org/10.64898/2026.10.08.757619; this version posted October 10, 2026. The copyright holder for this preprint (which was not certified by peer review) is the author/funder, who has granted bioRxiv a license to display the preprint in perpetuity. It is made available under a CC-BY 4.0 International license.

an individual lipid species, and each column represents a biological replicate. Color intensity indicates the relative abundance of each lipid across XH001/TM7x and XH001Δ*gdpd*$_{Xhp1}$/TM7x cocultures. Loss of *gdpd*$_{Xhp1}$ altered neutral lipid metabolism, resulting in changes in triacylglycerol and diacylglycerol profiles associated with LD regulation and cellular lipid homeostasis.

bioRxiv preprint doi: https://doi.org/10.64898/2026.10.08.757619; this version posted October 10, 2026. The copyright holder for this preprint (which was not certified by peer review) is the author/funder, who has granted bioRxiv a license to display the preprint in perpetuity. It is made available under a CC-BY 4.0 International license.

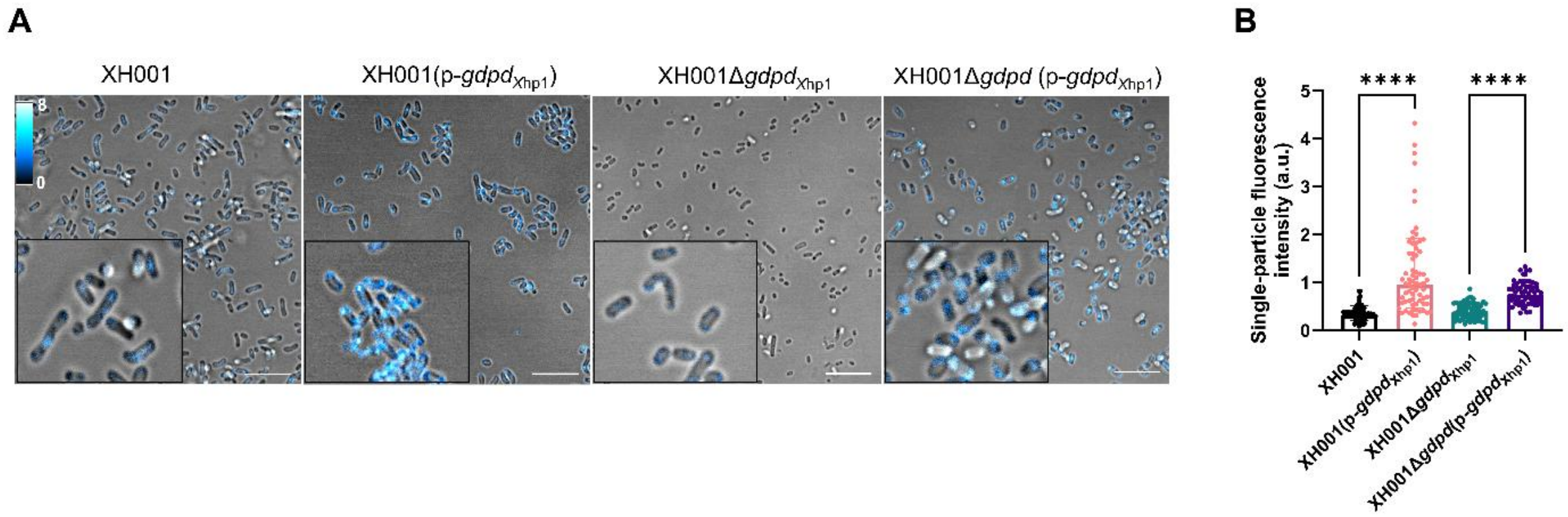

Supplementary Figure 3: Complementation with *gdpd*$_{\mathbf{Xhp1}}$ rescues LDs defects in the Δ*gdpd*$_{\mathbf{Xhp1}}$ mutant. (A) Representative confocal laser scanning microscopy images of LipidSpot-stained lipid droplets in WT XH001, XH001Δ*gdpd*$_{Xhp1}$, XH001(p-*gdpd*$_{Xhp1}$), and XH001Δ*gdpd*(P-*gdpd*$_{Xhp1}$) strains. Scale bar = 5 µm. (B) Quantification of LipidSpot fluorescence intensity in the indicated strains. Constitutive expression of *gdpd*$_{Xhp1}$ restored LD accumulation in both complemented strains, demonstrating that GDPD$_{Xhp1}$ is sufficient to drive LD formation independently of TM7x. Statistical significance was determined by one-way ANOVA with multiple-comparisons testing; ****, $P < 0.0001$.

bioRxiv preprint doi: https://doi.org/10.64898/2026.10.08.757619; this version posted October 10, 2026. The copyright holder for this preprint (which was not certified by peer review) is the author/funder, who has granted bioRxiv a license to display the preprint in perpetuity. It is made available under a CC-BY 4.0 International license.

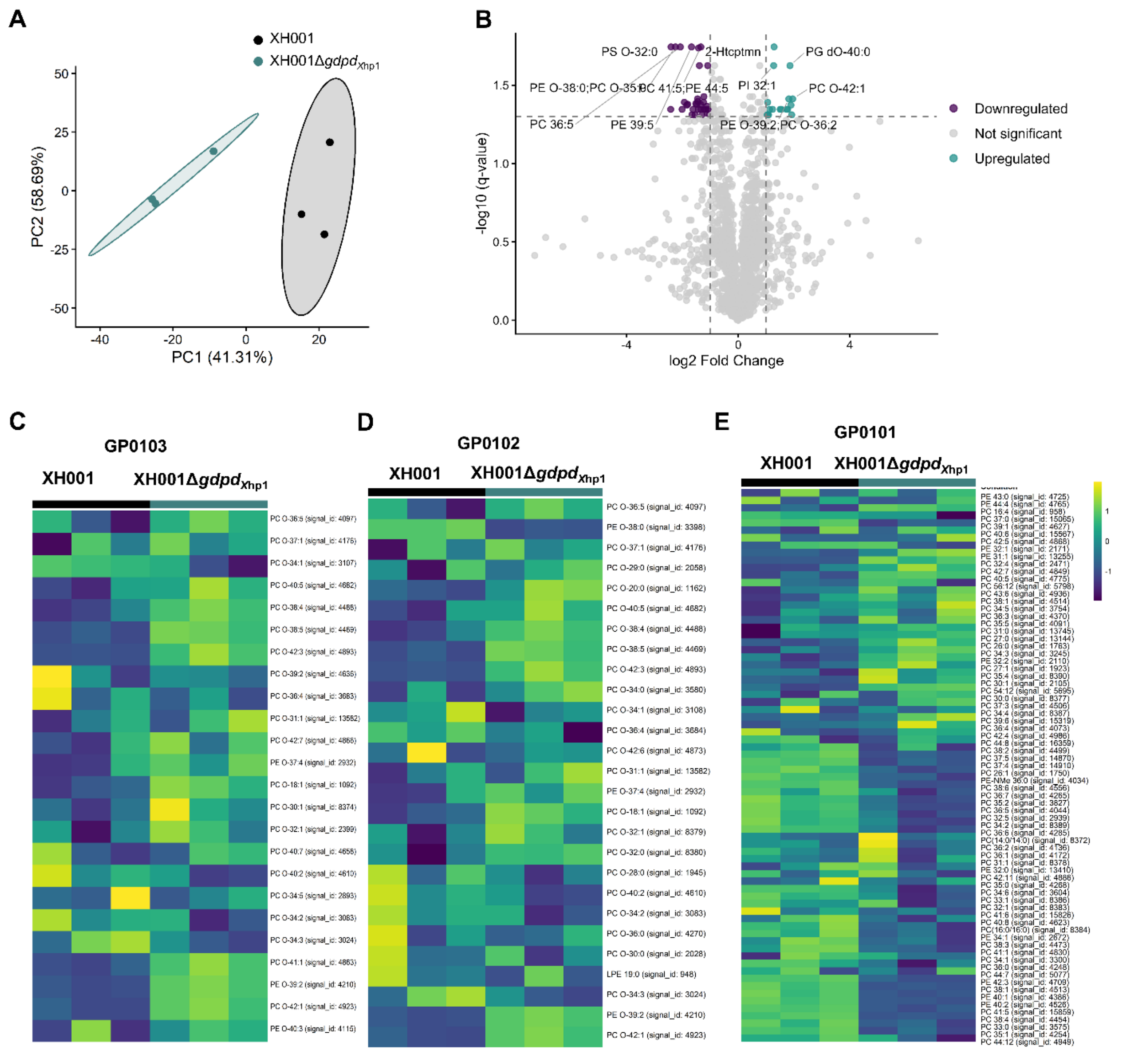

Supplementary Figure 4: Global lipidomic profiling reveals phosphatidylcholine remodeling in XH001Δ*gdpd*$_{\mathrm{Xhp1}}$ monoculture. (A) PCA of lipidomic profiles demonstrating clear separation between XH001 and XH001Δ*gdpd*$_{\mathrm{Xhp1}}$ monocultures, indicating distinct lipid compositions following deletion of *gdpd*$_{\mathrm{Xhp1}}$. Each point represents an individual biological replicate, and ellipses indicate the 95% confidence regions of each group. (B) Volcano plot showing differential lipid metabolite abundance in XH001Δ *gdpd*$_{\mathrm{Xhp1}}$ relative to XH001. Log$_2$ fold changes are plotted against adjusted *P* values (q values). Significantly decreased and increased metabolites are highlighted in purple and yellow, respectively. (C-E) Heatmaps showing differential abundance patterns of 1-(1Z-alkenyl),2-acylglycerophosphocholines [GP0103] (C), 1-alkyl,2-acylglycerophosphocholines [GP0102] (D), and diacylglycerophosphocholines [GP0101]

bioRxiv preprint doi: https://doi.org/10.64898/2026.10.08.757619; this version posted October 10, 2026. The copyright holder for this preprint (which was not certified by peer review) is the author/funder, who has granted bioRxiv a license to display the preprint in perpetuity. It is made available under a CC-BY 4.0 International license.

(E). Each row represents an individual lipid species, and each column represents a biological replicate. Color intensity indicates the relative abundance of each lipid species across XH001 and XH001Δ*gdpd*$_{Xhp1}$ monocultures. Deletion of *gdpd*$_{Xhp1}$ resulted in distinct phosphatidylcholine remodeling, characterized by increased ether-linked phosphatidylcholine species (GP0102 and GP0103) and reduced diacylglycerophosphocholine species (GP0101), indicating altered phospholipid homeostasis and membrane lipid composition.

bioRxiv preprint doi: https://doi.org/10.64898/2026.10.08.757619; this version posted October 10, 2026. The copyright holder for this preprint (which was not certified by peer review) is the author/funder, who has granted bioRxiv a license to display the preprint in perpetuity. It is made available under a CC-BY 4.0 International license.

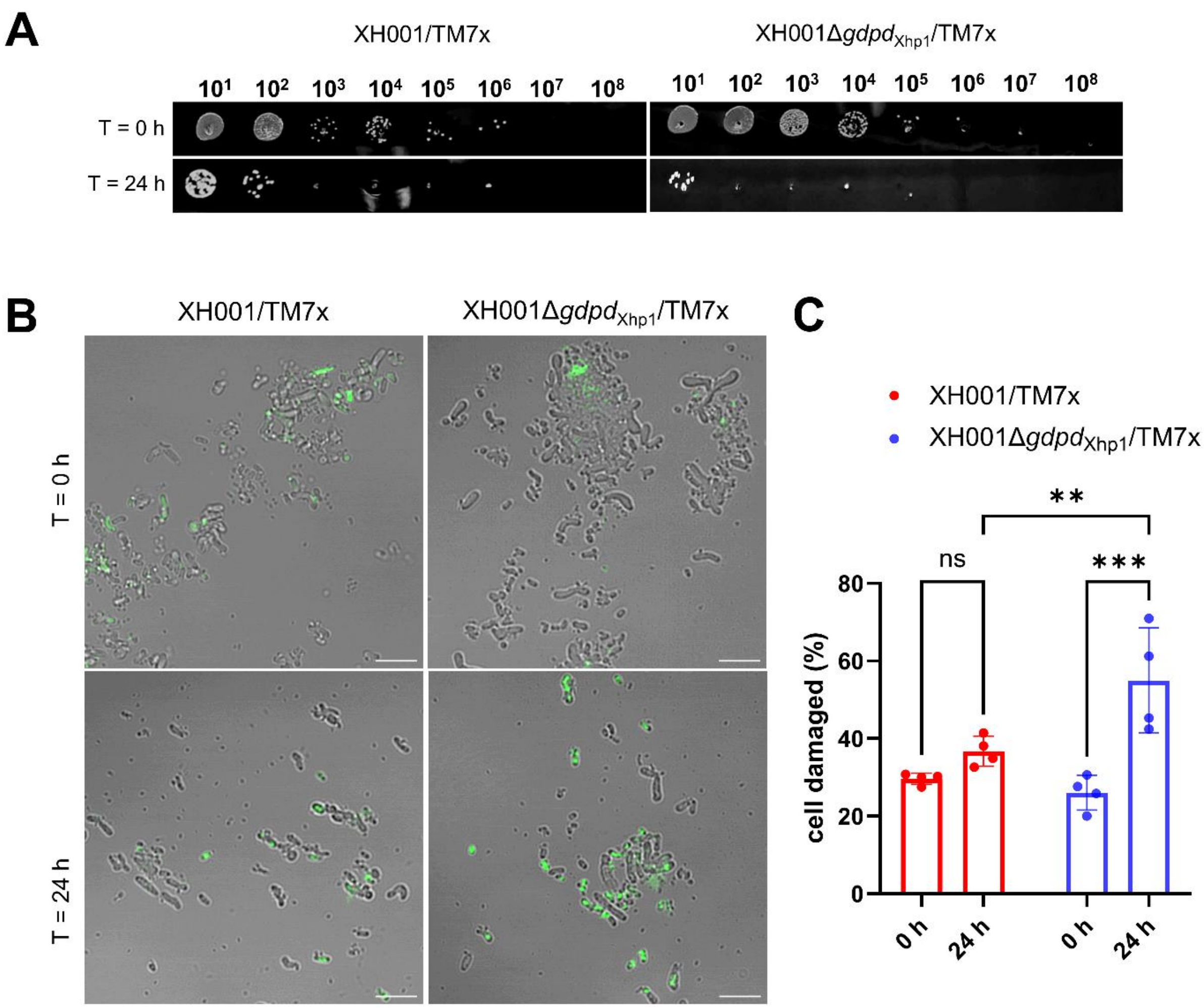

Supplementary Figure 5: Loss of GDPD$_{Xhp1}$ reduces survival and increases cell damage during nutrient starvation. (A) Representative spot-dilution assay of XH001/TM7x and XH001Δ*gdpd*$_{Xhp1}$/TM7x cocultures immediately after transfer to PBS (T = 0 h) and following 24 h incubation in nutrient-free PBS (T = 24 h). The Δ*gdpd*$_{Xhp1}$/TM7x coculture exhibited reduced recovery after 24 h compared with the WT coculture. (B) Representative Sytox Green fluorescence images of XH001/TM7x and XH001Δ*gdpd*$_{Xhp1}$/TM7x cocultures at T = 0 h and T = 24 h. Sytox Green-positive cells indicate membrane-compromised or damaged cells. Scale bars, 5 µm. (C) Quantification of Sytox Green-positive cells shown as the percentage of damaged cells at T = 0 h and T = 24 h. Statistical significance was determined using an unpaired two-tailed Student's *t*-test. ***P* <0.01; ****P* <0.001, ns = not significant.

bioRxiv preprint doi: https://doi.org/10.64898/2026.10.08.757619; this version posted October 10, 2026. The copyright holder for this preprint (which was not certified by peer review) is the author/funder, who has granted bioRxiv a license to display the preprint in perpetuity. It is made available under a CC-BY 4.0 International license.

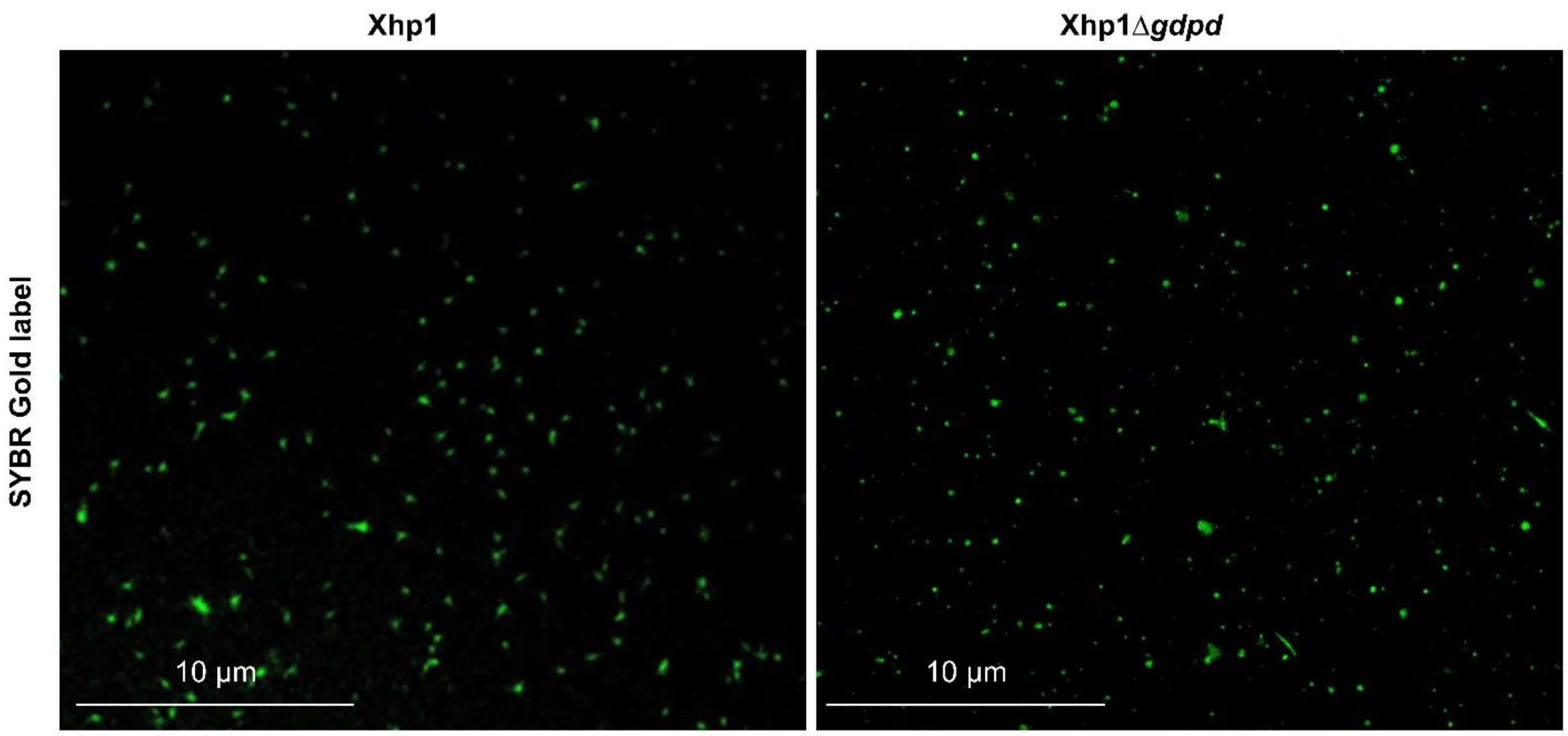

Supplementary Figure 6: Visualization of isolated Xhp1 and Xhp1Δ*gdpd* phage particles by SYBR Gold staining. Prophages were induced from mid-log-phase cultures of *S. odontolytica* XH001 (OD_{600} ≈ 0.5) with mitomycin C (0.01 μg mL^{-1}) for 3 h. Phage particles released from the induced cultures were recovered and purified, stained with SYBR Gold, and visualized by fluorescence microscopy. Representative images of WT Xhp1 (left) and Xhp1Δ*gdpd* (right) preparations are shown. Scale bars, 10 μm.

bioRxiv preprint doi: https://doi.org/10.64898/2026.10.08.757619; this version posted October 10, 2026. The copyright holder for this preprint (which was not certified by peer review) is the author/funder, who has granted bioRxiv a license to display the preprint in perpetuity. It is made available under a CC-BY 4.0 International license.

Supplementary Table 1: List of primers

Primer name	5'-3'
GDP_up_fwd	GCACCATATGAGCCTGCGCGGAGCCTGG
GDP_up_rev	GATTTAAGCCCCGATGCAGAAGGCCCCACC
GDP_CatP_fwd	TCTGCATCGGGGCTTAAATCAAGGAAAAATCC
GDP_CatP_rev	GTGGTCGCCCTTTCTACGGGGTCTGACG
GDP_dwn_fwd	CCCGTAGAAAGGGCGACCACCTGCACGC
GDP_dwn_rev	GCTCGAATTCCGAAGCCGGCACCGTCAAG
GDP_PCWU3_fwd	GCCGGCTTCGGAATTCGAGCTCGGTACC
GDP_PCWU3_rev	CGCGCAGGCTCATATGGTGCACTCTCAG
GDP_up check	CGAGCTACCTCCGAAGAGCG
GDP_dwn_check	GCGCGACCATCATCACCG
GDPD V1_fwd	GCAAATGGGTGTGGATGAGCTGACAATCTATCG
GDPD V1_rev	CGGATCCGCGCTACGGATGCACGCGAGATG
pET28a_V1_fwd	GCATCCGTAGCGCGGATCCGAATTCGAG
pET28a_V1_rev	GCTCATCCACACCCATTTGCTGTCCACC
GDPD_F_Check	GACGTGATCGGTATGGAG
pET28_F_Check	GCTAGTTATTGCTCAGCG
pJRD215_fwd	ATAATTCAGACCGTCTCGAGGCGAACCCCAGAG
pJRD215_rev	CGCGTGCATCCGTAGGGCGAAATGACCGACCAAG
gdpd_pJ215_fwd	GACTCTGGGGTTCGCCTCGAGACGGTCTGAATTATCAGACTG
gdpd_pJ215_rev	GCTTGGTCGGTCATTTCGCCCTACGGATGCACGCGAGATGGG
pJ_GDPD_check-F	GCGCGCGGTTACCGCACTATG
pJ_GDPD_check-R	GCCGCACAGCTCCATAGG
GDPD_XH_01395_F	GCTCGCTGATCGTCATTC
GDPD_XH_01395_R	GACGAATATCCGAGAACC
GDPD_XH_05820_F	CTGCTACGACGGTACTGG
GDPD_XH_05820_R	CGCATCAATGTTGAAGTAC
16S F	GGCCTTCGGGTTGTAAAC
16S R	CTGCTGGCACGTAGTTAG
